# Comparing written and photo-based indoor wayfinding instructions through eye fixation measures and user ratings as mental effort assessments

**DOI:** 10.16910/jemr.12.1.1

**Published:** 2019-01-09

**Authors:** Laure De Cock, Ralph Michels, Pepijn Viaene, Alain De Wulf, Kristien Ooms, Nina Vanhaeren, Nico Van de Weghe, Philippe De Maeyer

**Affiliations:** Ghent University, Belgium; Eyedog Wayfinding, Culemborg, The Netherlands

**Keywords:** Indoor navigation, wayfinding aids, route communication, eye tracking, attention, eye movement, head-mounted eye tracker, mental effort, usability

## Abstract

The use of mobile pedestrian wayfinding applications is gaining importance indoors. However, compared to outdoors, much less research has been conducted with respect to the most adequate ways to convey indoor wayfinding information to a user. An explorative study was conducted to compare two pedestrian indoor wayfinding applications, one text-based (SoleWay) and one image-based (Eyedog), in terms of mental effort. To do this, eye tracking data and mental effort ratings were collected from 29 participants during two routes in an indoor environment. The results show that both textual instructions and photographs can enable a navigator to find his/her way while experiencing no or very little cognitive effort or difficulties. However, these instructions must be in line with a user’s expectations of the route, which are based on his/her interpretation of the indoor environment at decision points. In this case, textual instructions offer the advantage that specific information can be explicitly and concisely shared with the user. Furthermore, the study drew attention to potential usability issues of the wayfinding aids (e.g. the incentive to swipe) and, as such, demonstrated the value of eye tracking and mental effort assessments in usability research.

## Introduction

The use of mobile pedestrian wayfinding applications (e.g. Insoft, MediNav by Connexient, SPREO Indoor Navigation, Meridian) is a form of wayfinding aid that is omnipresent outdoors and is gaining importance indoors, especially in very large and complex buildings. To enable an optimal use of these applications indoors, it must be examined how wayfinding information should be conveyed to the navigator in an user-friendly and adequate way [ 1].

Maps (with or without a route displayed on top) are frequently used to communicate a path from A to B. The survey perspective of the environment, that maps offer, enables a user to build up and improve his/her cognitive map (i.e. a mental representation of the external environment). However, the limited screen size decreases the map interaction quality in terms of efficiency and effectiveness and may result in more map reading difficulties [2]. Moreover, many visitors of complex buildings, especially non-recurrent visitors, wish to maximise the ease of wayfinding and have no interest in acquiring or improving their cognitive map [3].

A valuable alternative can be found in (simple) turn-by-turn route instructions, defined and generated by a system. Here, the route is divided into segments. In route instructions, these segments should be described by at least two elements, which form so-called view-action pairs. Firstly, a description must contain an indication of movement or state-of-being describing a wayfinding action, such as ‘turn left at’, ‘go down to’, ‘continue along’ and other basic motor activities. Secondly, a route (segment) description should also contain unambiguous and concise references to clearly visible physical features along the route or at decision points that serve as environmental cues to correctly pinpoint the location where that wayfinding action should take place and can act as feedback to the navigator [4, 5, 6]. These salient physical features are often referred to as landmarks. These play an important role in natural wayfinding behaviour as they are central to all forms of spatial reasoning (e.g. orientation, wayfinding) and spatial communication [7].

Often, one automatically assumes that these view-action pairs are expressed verbally or textually. However, a symbol (e.g. an arrow) combined with a photograph depicting (one or more landmarks at) a location may be equally as useful. In this explorative study, two mobile wayfinding aids are compared, in terms of cognitive load; one provides written route instructions (*SoleWay*) and the other photographic-based route instructions (*Eyedog*).

This paper is organised as follows. In the next section, previous work on indoor wayfinding smartphone applications and their use is described. Section 3 presents the study design. Following, the results and discussion are presented in sections 4 and 5 respectively. Finally, section 6 presents the main conclusions and future work on this topic.

## Background

According to Fallah, Apostolopoulos, Bekris and Folmer (2013), an indoor human wayfinding system should include at least four functionalities or components: (1) a (basic) form of localisation, (2) the ability to plan a path and turn it into easy to follow instructions, (3) the ability to retrieve and store different types of information and (4) the ability to interact with a navigator [8]. This paper only focusses on the last functionality, namely how a system can adequately interact with a user to provide the previously determined directions. More specifically, the user interaction of two indoor wayfinding smartphone applications, that are available to the public, will be compared and form the topic of this paper, namely *SoleWay* and *Eyedog (Indoor Navigation)*.

### SoleWay

**Figure 1.**
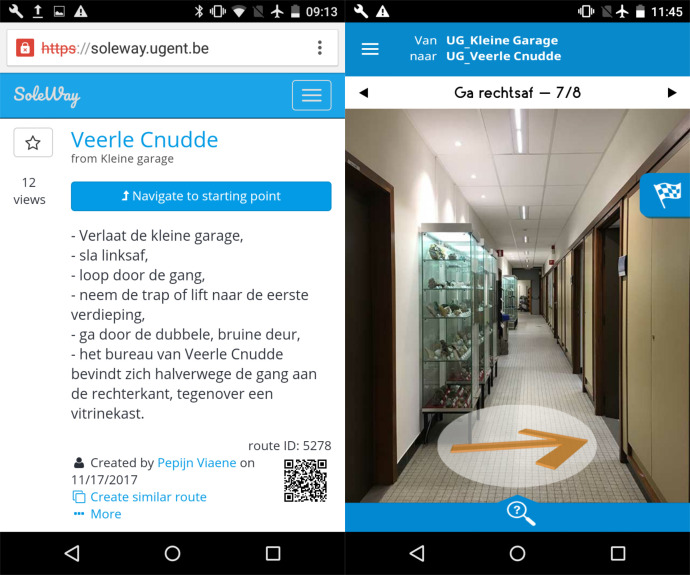
Screenshot SoleWay route (on the left) and screenshot Eyedog route (on the right).

The first, *SoleWay*, offers indoor wayfinding support through textual instructions (see Figure 1, on the left). The app and related website are based on a crowd-based outsourcing platform. As such, a community of (potential) wayfinders is created. On the one hand, people who are familiar with a certain route can add written route descriptions to the *SoleWay* database. Added route descriptions are purely textual. Each description is geographically located by pinpointing the approximate location of the starting point (e.g. main entrance) or the building in which the described route is situated on a map (i.e. Google Maps). On the other hand, wayfinders can find these descriptions by searching for the destination through a search box. The *SoleWay* platform will then provide the user with all descriptions of routes that lead to that destination and are in the vicinity of the user or the building of interest. Consequently, there is no need to spatially model the indoor environment or to use indoor positioning techniques which would require the installation of sensors (e.g. RFID tags, Bluetooth beacons, WLAN), which in turn may require costly infrastructure or augmentation of the building. A member of the community only needs a device with network capabilities (e.g. a smartphone). As a result, the cost and complexity of the application is minimised. *SoleWay* was developed by co-author Prof. Nico Van de Weghe (Department of Geography, UGent - https://soleway.ugent.be).

These textual instructions offer the advantage that (more) abstract concepts can concisely be conveyed to the user. Navigators have a good understanding of locations like the main entrance, a reception (desk) and a meeting room, regardless of their appearance or the extent to which they can change over time in terms of design. Consequently, these locations can easily be incorporated in an instruction without lengthy or detailed descriptions [9]. Analogously, it can be assumed that these textual route instructions (only) contain the most relevant information for the wayfinding task at hand and are not cluttered with unnecessary elements. In contrast, photographs may contain a high level of information and details as these depict everything present at a specific location.

Unfortunately, this is a double-edged sword as it can be difficult to determine what the most relevant, essential or suitable (type of) wayfinding information is. There are no unequivocal criteria for selecting salient physical features that can be used to describe the location where an action should take place as part of previously mentioned view-action pairs. Several studies have shown that landmarks are the most commonly used cues to enable wayfinding decisions and that route instructions containing landmarks as descriptive features are rated as highly effective [10, 11]. The main reason for this is that they allow fast reasoning and efficient communication for directing a person from A to B. Firstly, because they act as points of correspondence between different forms of spatial knowledge (e.g. reality, wayfinding tools [such as maps] and the cognitive model of the environment) [12]. Secondly, landmarks define a place with reduced representational complexity. While a place may exhibit a high level of information and details, a landmark is an anchor point that is abstracted to a node without internal structure [7]. As Streeter, Vitello and Wonsiewicz (1985) put forward, however, the landmark selection process is highly individual [13]. It depends on the perception and individual preferences of the observer, which are influenced by gender, age, social and cultural background, experience, familiarity with the environment and intentions [14]. For example, women prefer three-dimensional objects over two-dimensional elements [15]. In addition to the selection of the correct wayfinding information, the assessment of the adequate amount of information may be problematic as well. An instruction that is too brief may lead to uncertainty, while too much information can result in confusion and both will lead to higher cognitive load levels [16].

### Eyedog

The other smartphone application, *Eyedog*, provides wayfinding support by means of ‘street-view’ like photographic imagery (see Figure 1, on the right). The route is presented as a sequence of photographs wherein the user can (manually) swipe back and forth. The photographs are augmented with textual or schematic (e.g. arrows) directions to clarify the intended wayfinding instruction. Although *Eyedog* can operate in combination with indoor positioning systems, similar to *SoleWay* it can function without the use of external hardware. In contrast to *SoleWay*, the indoor environment is spatially modelled with the help of a network of nodes and edges attributed with weights and photographs. Based on this network, shortest paths are generated automatically. *Eyedog* was developed by co-author Ralph Michels (PhD researcher and CTO of *Eyedog Indoor Navigation -* http://www.eyedog.mobi).

Photographs, as used by *Eyedog*, represent the indoor environment at a certain location. This way, the landmark and wayfinding information selection process is to a large extent in the hands of the user. Photographs support this process as the visual sense contributes greatly to the recognition of landmarks and the estimation of distance and orientation during navigation [8]. Indoors this is of great value. Indoor routes are often characterised by frequent shifts in direction and, therefore, require a higher density of landmarks to be clearly described. Moreover, the number of object categories from which landmarks can be selected is usually limited indoors [17]. Accordingly, several studies have shown that, in comparison to paper and mobile maps, participants prefer images to visualise the environment while executing wayfinding tasks indoors as the use of photographs leads to improved wayfinding performance in terms of task duration and success rate [ 17, 18].

### Performance measures

The comparison between different modalities to convey route descriptions to a navigator is generally based on the time needed to complete a wayfinding task and whether or not a person is able to reach the destination with the help of a specific modality. In most of these indoor wayfinding studies, however, it is very rare that a person does not reach the destination point. Furthermore, the observed task duration differences may be statistically significant, but in practice these are very small. Other performance measures that may be more adequate to reflect the usability of a description are location and orientation accuracy [3], numbers of errors, feeling lost episodes and/or dwell points [19], smartphone interaction recordings to identify wayfinding strategies [20] and user ratings with respect to quality and usefulness [ 16].

Another research tool, which is relatively new in the domain of indoor wayfinding, is eye tracking. The analysis of gaze characteristics can provide useful insights regarding a navigator’s use of environmental and wayfinding information, and the interplay of both [21]. Consequently, in recent years eye tracking has frequently been used in a wide range of settings within the field of pedestrian navigation (e.g. spatial decision making, map interaction, wayfinding aids) [22]. However, the number of studies, specifically within the context of communication modalities of indoor pedestrian wayfinding systems, is limited.

Ohm, Müller and Ludwig (2017) used eye tracking measures as fixation time, number of fixations and revisits to the mobile phone screen to demonstrate that participants preferred a reduced interface displaying landmarks and simplified route segments instead of an interface using floor plans [23]. In contrast to the original experimental design, the smart phone screen was seen as a single area of interest. The small screen and the limited accuracy of the eye tracking device did not allow fixations to be attributed to different interface elements. Following, Schnitzler et al. (2016) investigated what the effect of a wayfinding aid (i.e. no map, paper map, digital map) was on fixation frequencies at decision points [21]. The number of fixations was determined for three areas of interest: signage, correct route option and incorrect option. Next, Li (2017) [18] used the number of fixations and their duration to create heat maps and gaze plots on photographs and maps to investigate the role of maps in combination with other aids during indoor wayfinding. In these three studies, eye tracking data was collected by a group of test persons that individually completed an indoor route to a destination with the help of a wayfinding aid. During this task, participants were equipped with a mobile eye tracker.

### Mental effort

Most studies on communication modalities of (indoor) pedestrian wayfinding applications focus on the usability of such a modality compared to or in combination with (mobile) maps. Moreover, no studies take into account the aspect of mental effort. Mental effort refers to the proportion of working memory capacity that is allocated to the (instructional) demands of the task and can be used as an index to assess the cognitive load that the execution of a task imposes on a person [24, 25]. Photographs and textual descriptions are both external representations of (wayfinding) information that are employed to support memory and thinking [9]. A person must translate such a representation (e.g. of a location), which is briefly stored in the short-term memory, to reality (e.g. the corresponding actual location in his/her surroundings). This translation requires the interaction of the short-term memory with previously acquired knowledge and skills (e.g. orientation skills) stored in the long-term memory. In turn, this interaction (i.e. working memory) and, as such, this translation demand mental effort [26]. Central in the cognitive load theory is that the working memory capacity is limited [27]. Visitors of large-scale spaces, especially first-time visitors, may already experience high stress levels and a significant working memory load caused by factors other than the wayfinding task. The wayfinding aid and the method used to present wayfinding information (e.g. words or images) can reduce the complexity of the decision-making process and therefore the cognitive load [28].

In general, there are four ways to assess mental effort: (1) indirect (performance) measures, (2) subjective measures, (3) secondary task measures, and (4) physiological measures [27]. In this study, subjective measures (i.e. rating scales) and physiological measures (i.e. eye tracking) will be used to assess which communication modality (i.e. written or photographic-based route instructions) requires less mental effort to understand and to act in accordance with. A large number of studies have used a nine-grade rating scale as a subjective measure to examine the experienced mental effort [24, 29, 30]. For an extensive overview of these studies, we refer to [31] and [24]. This frequent use has proven that the numerical values of a (nine-grade) rating scale enable test persons to veraciously express the required mental effort. Furthermore, multiple measurements during an experiment are possible. This way, a more detailed analysis of mental effort and task complexity variations can be conducted [27, 31].

Following, eye tracking can also be used as a measure of the processing demands of a task [32]. Especially in problem solving tasks, longer fixations and shorter saccadic amplitudes are linked to more effortful cognitive processing and indicate that a person has (more) difficulties in extracting information or relating this information to internalised representations. In scene perception, features that are considered more important, interesting or semantically informative generate longer fixations and more revisits compared to those elements that are perceived less important. Additionally, several studies assume that the number of fixations and saccadic rate overall is negatively correlated with the search efficiency and could be an indication of the difficulties a person experiences while collecting relevant information [ 33, 34].

Ooms (2016) emphasizes the importance of using mixed methods in usability research [35]. Using multiple eye tracking measures and mental effort ratings makes it possible to verify the results across datasets. This improves the reliability and validity of the study. However, some authors have argued that fixation measures and mental effort ratings measure different aspects of cognitive load [27, 32]. Fixations represent parts of the task or an individual problem, while an effort rating represents the mental effort of the overall task or process (e.g. the total number of problems). As such, these may result in non-equivalent assessments of the invested mental effort. By asking mental effort ratings at multiple intermediate points, the authors hope to minimise such distortion.

## Methods

Most user studies in interactive cartography are conducted in controlled, laboratory environments. Roth et al. (2017) state in their research agenda the need for both laboratory and field-based studies [ 36]. Explorative user studies in the field are essential to confirm laboratory findings, or to identify new aspects that need follow-up of laboratory research. Therefore, in order to assess the experienced mental effort linked to the textual route instructions offered by SoleWay on the one hand and Eyedog’s photographic-based instructions on the other hand, both apps are used in a real environment. Participants are guided by Eyedog on one route and by SoleWay on another route in a complex building. During the full extent of both routes eye fixations are recorded and user ratings on a nine-grade scale are collected at intermediate points. Four eye tracking measures are extracted (the number of revisits, fixation count, fixation time and average fixation duration) and two areas of interest are defined (smartphone screen and signage). Although all eye tracking measures are correlated, they do not measure the same aspect of mental effort. The number of revisits indicates how many times participants needed to switch their gaze from the environment to an aid (smartphone or signage). The average fixation duration indicates how difficult it is to interpret the information provided by one fixation on a specific element of the wayfinding aid, while the total fixation time and count indicate how difficult it is to gain the relevant wayfinding information from the wayfinding aid in general and translate this to the environment. To be able to interpret the eye tracking results correctly, all measures must be analysed together [33]. Saccadic measures are not included in the analysis but could be an equally valid alternative. After completing the two routes, participants answered a questionnaire to gain insight in their general appreciation of the two wayfinding aids.

### Participants

In total 14 male and 15 female subjects participated in the experiment. The questionnaire, wherein participants were asked to rate a series of statements, revealed the following. Participants were, on average, relatively familiar with (parts of) the test environment (see materials section). As such, they are acquainted with the building’s structure and design. In contrast, the destinations along the route were not known to them. Furthermore, test persons had used smartphone applications as wayfinding aid before outdoors, but rarely in an indoor setting. Their ages ranged between twenty and sixty years old ( *M* = 34, *SD* = 9). During the test, they were not distracted by the researcher following them or by the mobile eye tracking device. Five participants were excluded from the eye tracking results, because the tracking ratio was too low. The required tracking ratio was set to 95%.

### Materials

During the completion of both routes, participants wore a SMI ETG 2.1 mobile eye tracking device (60 Hz / 30 FPS). Fixations were calculated with the help of the SMI Event Detection (dispersion-based) algorithm and were transferred manually to four reference images (i.e. one for each route and application). Each reference image displayed two categories (i.e. (screen of) smartphone, signage (along the route)), which were attributed with areas of interest by using the semantic gaze mapping tool of BeGaze 3.6.

Both wayfinding apps (i.e. *Eyedog Indoor Navigation* 1.0.0 and *SoleWay* v15) were installed and presented on the same smartphone, namely an LG Nexus 4. As mentioned in section 2, *Eyedog* automatically provided the shortest routes between the selected destinations based on a network model of the building. The *SoleWay* routes were formulated and entered manually. A *SoleWay* route is a text depicted on a single screen. Each line in the text consisted of one view-action pair specifying the location and the required wayfinding action as simple as possible (see Figure 1). For each route, the number of lines was comparable to the number of photographs.

**Figure 2.**
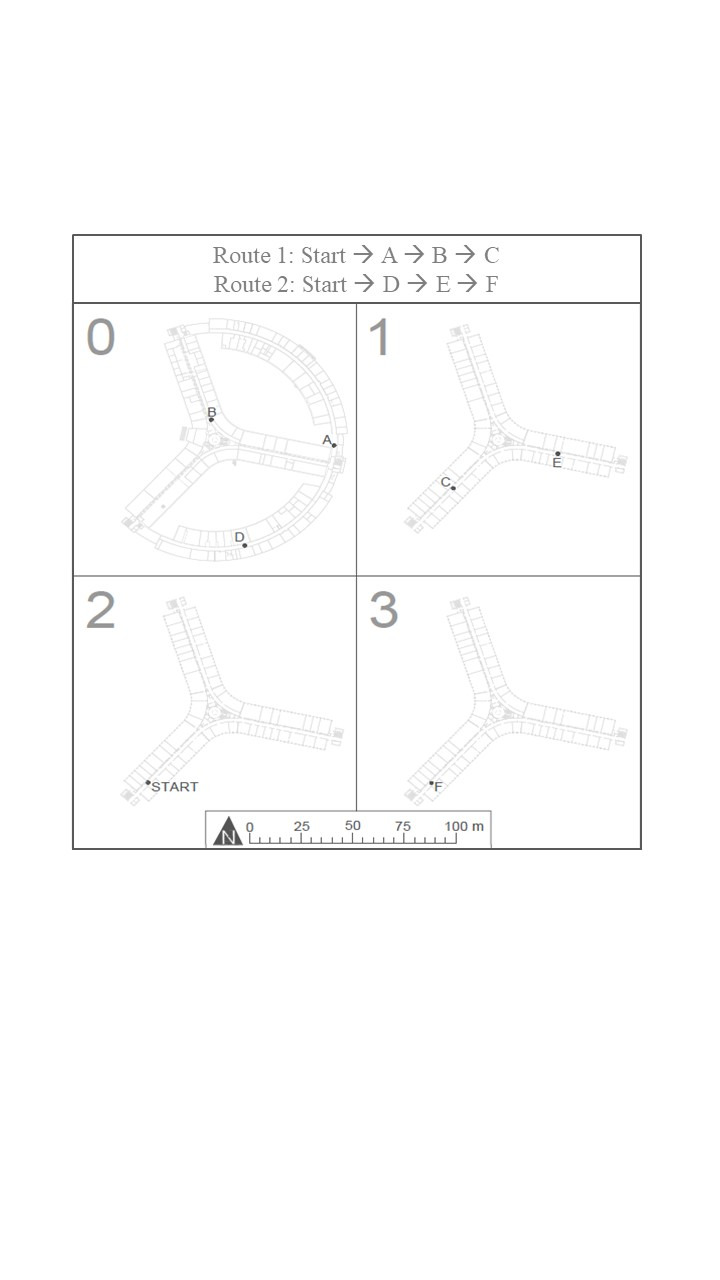
Illustration of the building and routes.

The star shaped building (see Figure 2) was considered to be fairly complex by most participants. It was built in 1976 and has a traditional interior (see Figure 1 and 4). Within this building two routes were selected. Both routes had had a total length of approximately 360 meters and consisted of three connected route segments leading to a destination. All participants completed the same routes (see Figure 2). The first route went from the starting point on the second floor level to the men’s toilet (destination A) on the ground floor. From there, participants were asked to go to the secretariat of the Marine Biology Research Group (B). Finally, they were asked to find the Geophysics Processing Lab (C) on the first floor level. The second route started at the same starting point. The first intermediate destination was the small garage (D) in the curved outer corridor on the ground floor level. The following destination was the office of prof. V. Cnudde on the first floor level (E). The final destination of the second route was lecture room 3.065 on the third floor (F). Route 1 includes 22 decision points and route 2 counts 24 decision points.

Finally, the following statements (subdivided in four categories) are rated on a seven-grade scale in the concluding questionnaire:

Evaluation of the route

*(1) “The route was complex.”*

**Evaluation of the wayfinding instructions

*(2) “I often had doubts about the further course of the* *route.” (3) “The wayfinding instructions were clear.” (4) “The wayfinding instructions were easy to follow.” (5) “The wayfinding instructions were detailed enough.” (6) “The wayfinding instructions were adequate to convey the route.”*

**Memory of the route

*(7) “After the experiment, I am able to complete the same route without help of a wayfinding aid.” (8) “After the experiment, I am able to verbalise route instructions to a person who is not familiar with the route.” (9) “After the experiment, I am able to draw the route on a floor plan.”*

**Application recommendation

*(10) “I would recommend the application for other buildings.”*

### Procedure

At the beginning of the experiment, the participants were instructed as follows. “*After the calibration of the eye tracking device, you will be asked to complete two routes while wearing the eye tracking device. This device will register your eye movements. I will follow while completing these routes. One route will be explained with the help of SoleWay. During the other route, you will be guided by Eyedog. Each route starts in this office and at the end of each route we will check if the eye tracking device is still correctly calibrated. Each route consists of a number of destinations or intermediate stops. At each stop, I will ask you to rate the mental effort that was needed to reach this destination with the help of the wayfinding application. You can rate this effort on a nine-grade scale: zero being very, very low and nine being very, very high. Then I will give you the next destination. After the completion of both routes, you will be asked to fill in a small questionnaire. During the experiment, you may always ask for help or clarification. I will intervene if you would get lost.*”

The experiment proceeded as described. Five point targets placed at approximately 1.5 meters were used to calibrate the eye tracking device. For all five points, the gaze error is corrected, making the calibration more accurate for every additional point. Participants always completed the routes in the same order. However, the wayfinding app used to guide a participant along a route was randomised in order to assess the potential influence of familiarity with the experimental setup. As such, half of the participants completed the first route with *SoleWay* and the second with *Eyedog*, while the other half first used *Eyedog* and then *SoleWay*. The guidelines as expressed by Holmqvist et al. (2011) were taken into account during calibration, instruction giving and route completion [33]. After completing the two routes, participants filled in the final questionnaire.

### Data analysis

The significance of potential differences in eye tracking measures between both groups (i.e. *SoleWay* users and *Eyedog* users) was determined by a parametric test (see Table 2). As there is disagreement about whether (ordinal) rating scale data should be analysed with parametric statistics or nonparametric statistics [37], the normality of the mental effort and questionnaire data was tested with the Shapiro-Wilk Test. Because not all data samples are normally distributed, both the parametric t-test and the non-parametric Mann-Whitney test are conducted to determine the significance of potential differences between both *SoleWay* users and *Eyedog* users regarding mental effort. Both tests showed the same result and are listed in Table 1. The significance of potential differences between *Soleway* and *Eyedog* in the general questionnaire is also analysed with both a parametric and a non-parametric test, for the same reason. In this case, a Wilcoxon signed-rank test and a dependent t-test is executed, because all participants used both *Soleway* and *Eyedog* and are therefore in both groups. The results of both tests are indicated in Table 3.

**Table 1:** Overview of mental effort ratings at intermediate destinations for both routes^*^

	*SoleWay*			*Eyedog*				
**Route 1^c^**	*N*	*Mean*	*SD*	*N*	*Mean*	*SD*	*Sig*. ^a^	*Sig*. ^b^
Start - A	15	1.93	1.58	14	2.21	1.85	.771	.665
A - B	15	2.13	1.51	14	1.64	1.28	.369	.352
B - C	15	3.07	1.67	14	2.86	1.96	.532	.760
**Route 2^c^**								
Start - D	14	2.29	1.73	15	5.93	2.12	**.000**	**.000**
D - E	14	1.57	1.02	15	4.73	2.25	**.000**	**.000**
E - F	14	1.50	1.40	15	4.40	2.67	**.004**	**.001**

^*^ based on scores on a nine-grade scale. ^a^ two-tailed significance value at the 95 % confidence level resulting from a Mann-Whitney test. ^b^ two-tailed significance value at the 95 % confidence level resulting from an independent samples t-test with tested equal variances. ^c^ more information on route segmentation can be found in Materials section.

**Table 2:** Overview of eye fixation measures during route completion

	Route	Smartphone AOI					Signage AOI				
		*SoleWay*		*Eyedog*			*SoleWay*		*Eyedog*		
		*M*	*SD*	*M*	*SD*	*Sig*.*	*M*	*SD*	*M*	*SD*	*Sig*.*
Revisits	1	21.20	10.69	71.93	25.32	**.000**	8.11	6.31	4.43	2.68	**.003**
	2	32.50	8.33	66.70	23.99	**.000**	10.07	4.25	13.40	4.79	.096
Fixation Count	1	262.70	146.23	464.43	146.08	**.003**	21.80	14.97	11.21	6.86	**.029**
	2	291.71	64.57	490.60	197.46	**.002**	29.93	13.08	39.70	16.44	.138
Fixation Time [ms]	1	65,383.7	39,161.14	123,871.1	49,979.70	**.004**	5,170.9	3,084.99	2,572.9	1,864.47	**.033**
	2	76,494.0	18,717.71	122,391.1	52,893.86	**.006**	10,461.2	4,495.02	12,010.2	4,289.12	.403
Fixation Time [%]	1	24.91	9.75	37.939	11.04	**.006**	1.77	1.02	0.82	0.58	**.020**
	2	24.44	5.37	30.052	10.49	.146	3.27	1.19	3.07	1.16	.677
Average fixation duration [ms]	1	243.65	32.97	260.62	31.98	.223	214.57	107.55	211.15	61.57	.929
	2	262.92	36.73	248.12	17.35	.251	351.05	79.87	311.37	62.32	.186

* two-tailed significance value at the 95 % confidence level resulting from an independent samples t-test with tested equal variances

**Table 3:** Overview of statement ratings in questionnaire for both applications^*^

	*SoleWay*			*Eyedog*				
Statement^c^	*N*	*Mean*	*SD*	*N*	*Mean*	*SD*	*Sig*. ^a^	*Sig*. ^b^
(1)	29	-1.76	1.57	29	-1.14	1.68	.107	.065
(2)	29	-1.76	1.33	29	0.07	2.10	**.000**	**.000**
(3)	29	2.07	0.80	29	0.48	1.98	**.001**	**.000**
(4)	29	2.03	0.82	29	0.66	1.95	**.002**	**.002**
(5)	29	2.14	0.92	29	0.55	1.88	**.001**	**.000**
(6)	29	2.43	0.67	29	0.86	1.64	**.000**	**.000**
(7)	29	0.10	1.74	29	0.21	1.86	.586	.621
(8)	29	-0.21	1.78	29	-0.28	1.89	.747	.805
(9)	29	-0.41	1.76	29	-0.14	1.68	.437	.318
(10)	29	1.34	1.26	29	0.55	1.35	**.019**	**.018**

^*^ based on scores on a seven-grade scale. ^a^ two-tailed significance value at the 95 % confidence level resulting from a Wilcoxon signed-rank test. ^b^ two-tailed significance value at the 95 % confidence level resulting from a dependent t-test. ^c^ Overview of statements in the research design.

## Results

The results for the mental effort ratings can be found in Table 1. Participants experienced a significantly lower mental effort when using *SoleWay* while completing the second route.

Following, Table 2 shows the results for the eye fixation measures. Based on the number of revisits, fixations and fixation time, it can be said that SoleWay users spent more attention on the available signs along route 1, while Eyedog users focussed more (frequent) on the smartphone screen. With respect to the second route, Eyedog users still fixated more on the application, but there was no significant difference with respect to signage use. There are no significant differences between the average fixation duration on smartphone and signage. Finally, the results of the questionnaire were analysed analogously to the mental effort ratings (see Table 3).

## Discussion

An explorative study was conducted to examine different modalities to share wayfinding information by comparing two pedestrian indoor wayfinding applications, namely *Sole-Way* and *Eyedog*, in terms of mental effort. To do this, eye tracking data and mental effort ratings were collected from participants during a series of wayfinding tasks in an indoor environment.

No significant differences between the two applications were found with respect to the mental effort ratings collected during the first route and the experienced mental effort was relatively low (mostly less than 3 on the nine-grade scale). In contrast, participants had significantly more difficulties to understand, interpret and act in accordance with the view-action pairs displayed by photographs (i.e. *Eyedog*) during the second route. An explanation for this finding might be found in the eye tracking results.

The average fixation duration does not differ significantly. Therefore, the difficulty lies not in the interpretation of the information provided by one fixation on the wayfinding aids (smartphone and signage). The total fixation count and overall fixation time, however, show that *Eyedog* users looked significantly more to different elements of the smartphone screen during both routes. This seems logical as more information is displayed by the *Eyedog* interface. Users needed to interpret this information and relate the (selected) depicted features to reality. The number of revisits indicates that Eyedog users switched their gaze back to the smartphone more often, which indicates that information translation to the environment was more difficult compared to the text-instructions. More striking is the use of signage during the wayfinding tasks. During the first route, *SoleWay* users gave significantly more attention to signs along the route compared to *Eyedog* users. Although not statistically significant, this observation was turned around during the second route as a result of an increase in fixations on signs by *Eyedog* users. Nevertheless, also in this case the average fixation duration was not found to be significantly different.

The extent to which signage is fixated on can be related to (1) the availability of signage, to (2) the wayfinding task complexity and (3) whether or not the smartphone application provides sufficient information to ensure a comfortable wayfinding experience. Firstly, although the entire building has a similar design, a slightly larger amount of signs was visible along route two. This may have accounted for the increased use of signage for both applications during the second route compared to the traversal of the first route. However, the signage was the same for both wayfinding applications and, therefore, this cannot explain the substantial increase of attention to signs when using *Eyedog*. Secondly, the results of the questionnaire show that no significant difference was found in terms of experienced route complexity. As such, it is not expected that this factor influenced sign usage. Therefore, the finding with respect to signage is most likely to be explained by the third factor: a lack of (an) adequate (amount of) information offered by the application. For example, the conciseness of the written route instructions might have prompted wayfinders to collect additional information (through signs) in the first route. As mentioned in the introduction, the selection of the adequate amount of information can be challenging. With respect to *SoleWay*, this assessment has to be made by the author each time he/she describes a route. Turning back to *Eyedog*, if *Eyedog* did not provide sufficient or adequate wayfinding information during the second route, then this will have forced *Eyedog* users to rely more on signage while completing this second route. In turn, having to interpret both detailed photographs and a large number of signs could have led to the increased mental effort ratings as mentioned earlier.

Based on the recordings, it is clear that all *Eyedog* users that ostensibly experienced wayfinding difficulties (e.g. errors, doubt), encountered them at two specific decision points along route two.

**Figure 3.**
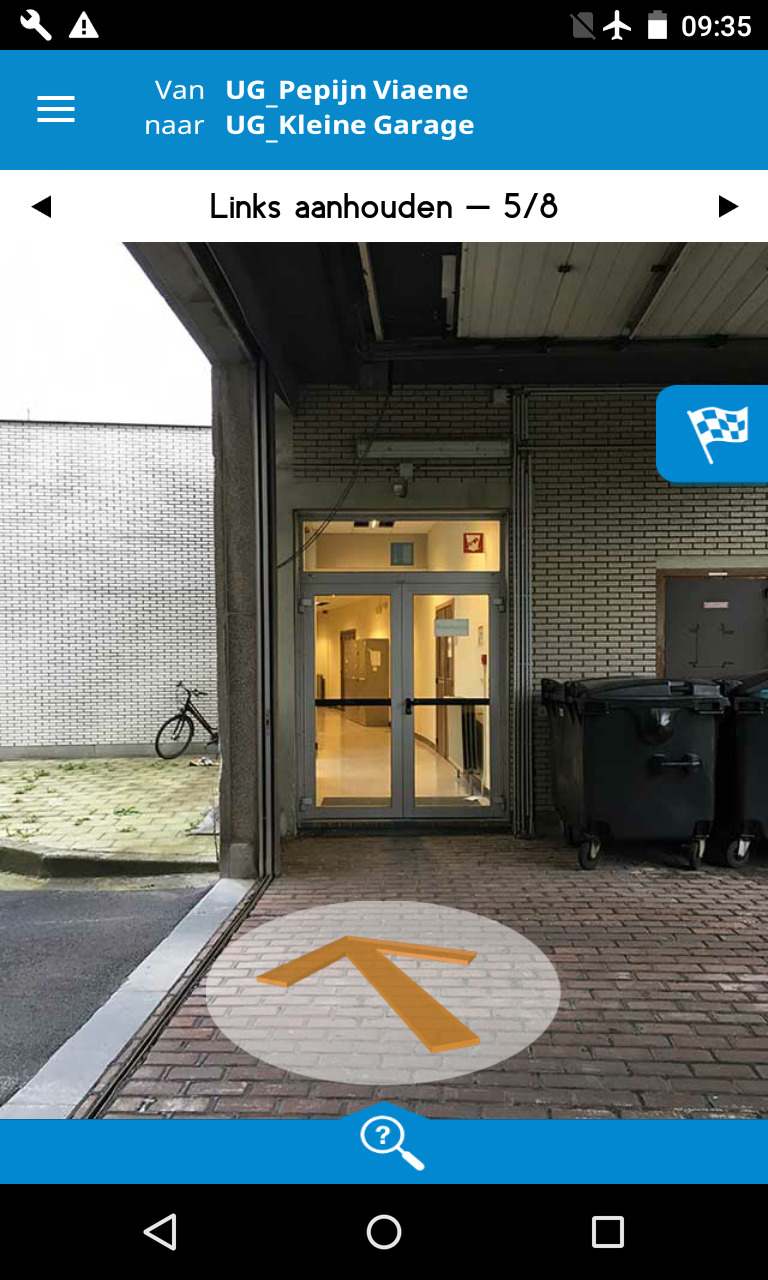
Screenshot Eyedog at start curved hallway.

Firstly, when participants arrived on the ground floor on their way to destination D, they came across a covered passageway that is part of the curved hallway where the destination is situated. At this passageway, however, participants were not aware that this hallway is curved. The *SoleWay* instruction, which was generated by a person based on his/her wayfinding experience, says “continue straight ahead through the glass double doors in front of you”. In contrast, the *Eyedog* platform took this curve into account when automatically generating wayfinding instructions as it starts from the spatial network of the building, which in turn is based on the floor plan of the building. As a result, the *Eyedog* instruction displays an arrow that is in line with the curve and mentions to “keep left” (see Figure 3). Although this is not incorrect, it was not in line with the expectations of the user. Consequently, there was much doubt whether to continue through the glass doors or to take a left turn to the inner courtyard.

Secondly, to pinpoint destination E as (intermediate) destination, *SoleWay* (see Figure 1) and *Eyedog* (see Figure 2) both refer to a display cabinet, which is situated right in front of the office. However, *SoleWay* specifies that this cabinet is located “halfway through the hallway”. This addition turned out to be of great value as a nearly identical cabinet is located at the beginning of the hallway. As a result, *Eyedog* users expected the destination to be (near the display cabinet) at the beginning of the corridor. In this case, the cabinet functioned as a ‘false landmark’, namely an identical or very similar object that can mislead the navigator as it is wrongfully associated with specific wayfinding actions [38]. Although several details on the photograph allow a differentiation between both cabinets, most participants only focus on the cabinet itself. As they are not familiar with the route, it is difficult for them to assess to what level of detail the depicted information needs to be interpreted.

At these two problematic decision points, *SoleWay* offered the advantage that the information had been interpreted in advance by the author of the instructions who is highly familiar with the route(s). As such, the author formulated route instructions that were (likely to be) in line with the user’s expectations and (unwittingly) differentiated between the cabinets by providing information regarding location in the hallway. This explains why *Eyedog* users experienced more mental effort compared to *SoleWay* users during the second route, as mentioned earlier. Analogously, the results of the questionnaire (see Table 3) show that they had less doubts about the further course of the route and found the *SoleWay* instructions significantly clearer, easier to follow, more detailed and more adequate to convey the route. Consequently, participants were more inclined to recommend *SoleWay* for other buildings than *Eyedog*.

Additionally, the recordings reveal a point of particular interest in terms of the usability and interface of *Eyedog*, namely the (lack of) incentive to swipe from one photograph to another. The apparent difference between *Eyedog* users that experienced little or no difficulties on the one hand and those that expressed high mental effort ratings on the other hand was that the first swiped freely between photographs. For example, these participants started by viewing the first three photographs or even the entire route before commencing the route itself and returning to the first photograph. That way they gained route knowledge, enabling them to connect different landmarks into a route. In contrast, the latter strictly focussed on a single photograph and only swiped to the following once they were absolutely sure that they had encountered the location depicted on that photograph. This group could therefore only rely on landmark knowledge for orientation in the building. Navigators are more successful in finding destinations inside a building when using multiple types of spatial knowledge, such as landmark knowledge and route knowledge [39]. As a result, the participants with solely landmark knowledge were not able to anticipate certain wayfinding actions and/or adapt their expectations with respect to the continuation of the route. In the current design, the app itself gives no clear incentive to swipe. At present, the user interface of *Eyedog* is being redesigned whereby the app will indicate within which distance a user is expected to swipe to the next picture. The discovery of these usability issues is an important advantage of qualitative, field-based research [ 36].

## Limitations

An explorative study was conducted to compare two pedestrian indoor wayfinding applications. Although explorative studies have many strengths, they also impose a few limitations. One of them is the difficulty to generalize findings as two existing systems were used in a realistic setting with possible end-users. This means that the configuration of the wayfinding aids, the architecture of the building and the familiarity of the participants with the building had an influence on the results. However, because the participants were not familiar with the destinations, both routes were equally new to them. Therefore, the order of the routes was not randomized. Another factor that caused some restrictions is the use of a mobile eye tracker. Extraction of saccadic measures can be difficult as both the eye and the head are moving. Therefore, this research used fixation measures and revisits as a measure for cognitive load.

## Conclusions and Future Research

This explorative study made an effort to gain more insight into the use of textual and photo-based route instructions by comparing two wayfinding aids in terms of mental effort. A combination of eye fixation measures and subjective user ratings showed that both textual instructions and (augmented) photographs can enable a navigator to find his/her way while experiencing no or very little cognitive effort or difficulties. However, certain decision points during a given wayfinding task require a specific interpretation of the situation or location to facilitate a comfortable wayfinding experience. In this case, textual instructions offer the advantage that this specific information can be explicitly and concisely shared with the user, providing that the author is able to deduce this information based on his/her wayfinding experience. Furthermore, the study drew attention to potential usability issues of the wayfinding aids and, as such, demonstrated the value of eye tracking and mental effort assessments to facilitate a user-centered design.

Future research will examine whether a new design, whereby incentives to swipe are given, can avoid the type of problems that were encountered in this study. This may also require an analysis of the swiping behavior of Eyedog users to examine when and where wayfinders need new information to get from A to B. This need for new information, hence a wayfinding instruction, can differ when using a Location Based System (LBS) instead of swiping. As already mentioned in the background section, Eyedog can operate with an LBS which facilitates this research topic. Another possibility for future research is linking different types of route instructions (e.g. text, image, video) to building architecture. To reduce the cognitive load during wayfinding, the right amount of information has to be provided in the most suitable manner. In other words, the right type of route instruction must be given at a certain type of decision points.

## Ethics and Conflict of Interest

The author(s) declare(s) that the contents of the article are in agreement with the ethics described in http://biblio.unibe.ch/portale/elibrary/BOP/jemr/ethics.html and that there is no conflict of interest regarding the publication of this paper.

## Acknowledgements

This research was supported and funded by the Research Foundation Flanders (FWO) ( http://www.fwo.be) by a PhD fellowship grant (FWO17/ASP/242).
